# Dynamics Govern Specificity of a Protein-Protein Interface: Substrate Recognition by Thrombin

**DOI:** 10.1371/journal.pone.0140713

**Published:** 2015-10-23

**Authors:** Julian E. Fuchs, Roland G. Huber, Birgit J. Waldner, Ursula Kahler, Susanne von Grafenstein, Christian Kramer, Klaus R. Liedl

**Affiliations:** 1 Institute of General, Inorganic and Theoretical Chemistry, University of Innsbruck, Innrain 82, 6020 Innsbruck, Austria; 2 Centre for Molecular Informatics, Department of Chemistry, University of Cambridge, Lensfield Road, Cambridge CB2 1EW, United Kingdom; Universite de Sherbrooke, CANADA

## Abstract

Biomolecular recognition is crucial in cellular signal transduction. Signaling is mediated through molecular interactions at protein-protein interfaces. Still, specificity and promiscuity of protein-protein interfaces cannot be explained using simplistic static binding models. Our study rationalizes specificity of the prototypic protein-protein interface between thrombin and its peptide substrates relying solely on binding site dynamics derived from molecular dynamics simulations. We find conformational selection and thus dynamic contributions to be a key player in biomolecular recognition. Arising entropic contributions complement chemical intuition primarily reflecting enthalpic interaction patterns. The paradigm “dynamics govern specificity” might provide direct guidance for the identification of specific anchor points in biomolecular recognition processes and structure-based drug design.

## Introduction

Cellular signaling critically depends on biomolecular recognition processes [1]. Understanding these processes on the molecular level is key for a comprehensive picture of living organisms. Models of biomolecular interactions evolved from first mechanistic explanation through Fischer's lock-and-key model that presumes static steric complementarity between the binding partners [2] and neglects any dynamic processes in the interacting entities. Koshland introduced dynamic aspects in the induced fit model which assumes that binding partners adapt their respective conformations to a state of maximum complementarity [3]. However, proteins undergo conformational transitions even in absence of binding partners existing as an equilibrium of conformations [4]. The conformational selection paradigm proposes that binding partners select the most appropriate conformation from this pre-existing ensemble of conformations [5]. Upon complex formation, equilibrium populations are shifted and a weakly populated state might become dominant [6]. Recently, conformational selection has become apparent in most biomolecular recognition processes [7].

Proteases provide prototypic protein-protein interfaces [8], binding and proteolytically cleaving peptides and proteins at a catalytic cleft [9]. The sub-pocket interactions of cleaved substrates (“degradome”) [10] are classified following the convention of Schechter and Berger [11]. Protease sub-pockets are numbered according to the corresponding substrate binding site over all sub-pockets Sn-Sn', with the peptide's scissile bond being the bond between N-terminal P1 and C-terminal P1'. Owing to a multitude of experimental techniques [12], substrate data is available for a wide range of proteases, e.g., via the MEROPS database [13]. Substrate information can be utilized for direct comparison of substrate recognition [14,15] and quantification of specificity [16]. Using these techniques, specificity within a protease binding site can be identified and visualized. In the well-characterized family of serine proteases substrate specificity originates primarily from interactions N-terminal to the cleavage site (non-prime side) [17], but also via remote exosite interactions [18,19]. Several studies aim at identifying a suitable binding paradigm and suggest conformational selection as most likely model [20,21]. Thrombin is a trypsin-like serine protease and key enzyme in the blood-clotting cascade [22,23]. On a structural level, active thrombin consists of a heavy and a light chain that is formed by proteolytic cleavage from a single precursor chain [24]. Thrombin includes several highly dynamic segments such as the autolysis loop (γ-loop) that is frequently missing in X-ray structures. The dynamic rearrangement of the active site of thrombin plays a role during zymogen activation via prethrombin-1 and prethrombin-2 as well as upon substrate binding [25]. As thrombin exists in two different states, exhibiting different biological roles, allosteric communication mediating the transition between the two forms plays an important role [26]. Thereby, binding of a Na^+^ ion switches the enzyme from the slow to the fast form which includes reordering of bound water molecules [27, 28]. Trypsin-like serine proteases are generally regulated via conformational plasticity around the substrate binding site, thus leading to the E*/E equilibrium [29]. The E* form is basically inactive towards substrate and Na^+^ binding and shows a collapse of the 215–217 ß-strand into the active site. In the active E form the S1 pocket is accessible and presents a negatively charged aspartate side-chain [30]. Direct P1-S1 interactions of the substrate with this amino acid explain the strong preference of thrombin for positively charged substrate residues (especially arginine residues) at P1 (C-terminal to the scissile bond). Further requirements have also been described for flanking amino acids [31,32].

However, differences between the other sub-pockets are smaller and less obvious from an enthalpic point of view. Broad literature highlights complex interplays between dynamics, solvation and ligand binding in thrombin [33,34,35]. We decipher molecular origins for the different degrees of specificity within sub-pockets of thrombin based on flexibility. Our analyses are based on two central concepts: We apply information theoretic cleavage entropy values [16] based on experimental substrate data to quantify local protease specificity and compare emerging specificity patterns to local binding site flexibility that is quantified via dihedral entropies [36] over backbone torsions derived from microsecond scale molecular dynamics simulations. In contrast to flexible sub-pockets, we expect rigid regions in interfaces to provide excellent anchor points for drug design as they allow for specific drug-target interactions. Locating appropriate interaction centers will support structure-based drug design targeting classical active sites [37] as well as allosteric pockets [38]. We expect our analysis of dynamic binding site features to be especially helpful in rational drug design on challenging protein-protein interactions [39].

## Results and Discussion

**Fig 1:**
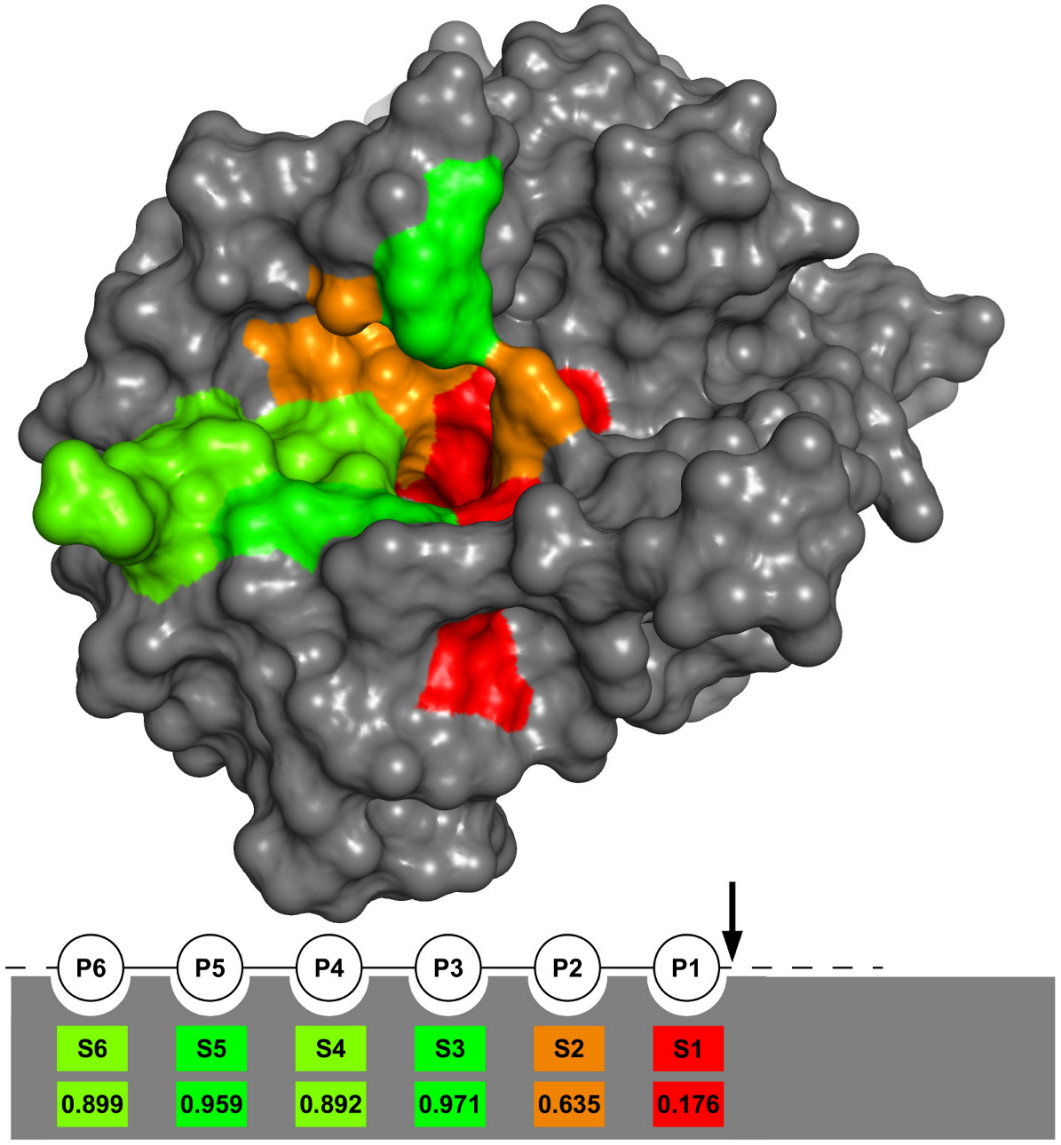
Cleavage entropy mapped to non-prime side thrombin sub-pockets. Mapping of cleavage entropy to thrombin sub-pockets shows S1 sub-pocket as most specific sub-pocket with a cleavage entropy <0.2, followed by the S2 sub-pocket with a cleavage entropy <0.7. Sub-pockets S3-S6 show little to almost no specificity with values for the cleavage entropy between 0.89 and 0.97.

We quantified thrombin sub-pocket specificity as cleavage entropy [16] based on substrate data from MEROPS [13] (see Fig 1). A value of zero represents absolute specificity for a single amino acid whereas absence of substrate preference in a sub-pocket is indicated via a cleavage entropy of one.

**Fig 2:**
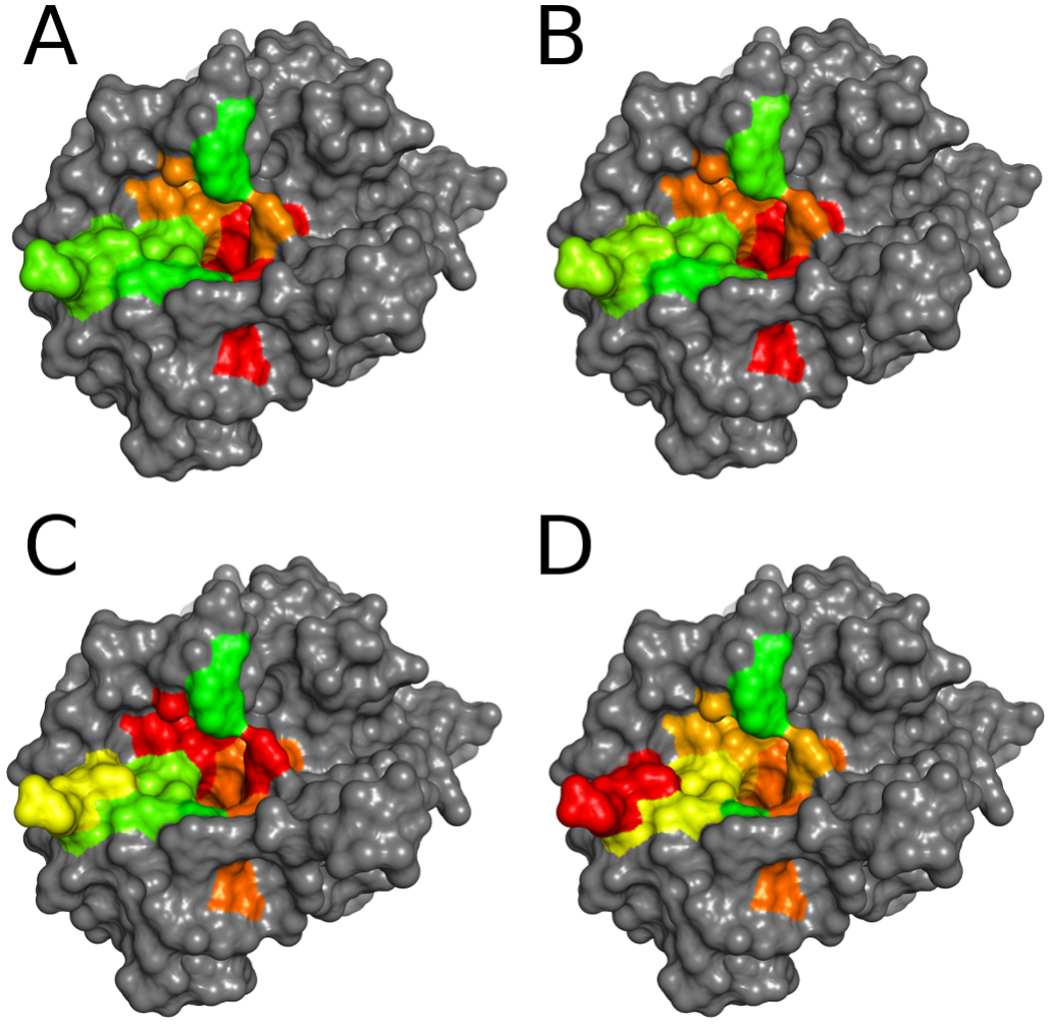
Flexibility landscapes of the thrombin binding site in comparison with cleavage entropy. Cleavage entropy mapped to thrombin sub-pockets (A) with colors ranging from red (specific) to green (unspecific) in comparison with sub-pocket average global backbone B-factors (B), φ entropy (C) and ψ entropy (D) with colors ranging from red (rigid or low dihedral entropy, respectively) to green (flexible or high dihedral entropy, respectively). Flexibility metrics in (B) and (C) show good correlation with (A). In contrast to other flexibility metrics, the S6 sub-pocket appears rigid according to ψ entropy (D). See Figure A in S1 File for a mapping based on ranks rather than absolute values.

To investigate the effect of conformational dynamics and thus conformational selection on substrate recognition, we simulated protein dynamics of unbound and fibrinogen-complexed thrombin for 1μs. To probe intrinsic local dynamics of the binding site region of thrombin without ligand, we calculated residue-wise backbone flexibility in holo state using Cα B-factors after a global alignment. We refer to holo systems for proteins that were created by artificially removing a ligand from a complex structure in contrast to a native apo structure. We grouped the global backbone flexibility into respective sub-pockets S6-S1, yielding average flexibilities of thrombin sub-pockets (see Table 1). Mapping to the binding site region gives a visual impression of intrinsic dynamics of the thrombin substrate binding cleft (see Fig 2).

**Table 1:** Sub-pocket-wise global backbone B-factors for holo simulations (Global Cα) and dihedral entropy values Sφ and Sψ. Sub-pocket average global backbone B-factors, Sφ and Sψ show S1 and S2 sub-pockets as the most rigid sub-pockets. Sub-pockets S3-S6 show varying flexibilities.

	S6	S5	S4	S3	S2	S1
Global Cα [Å^2^]	44.6	56.5	47.7	50.8	24.8	15.6
Sφ [J/(mol*K)]	34.6	35.9	35.6	36.6	32.5	33.3
Sψ [J/(mol*K)]	32.3	34.2	34.1	35.8	33.5	33.0

As expected from its buried location in the center of the protease, the S1 pocket was found to be the most rigid part of the binding site on a global scale. The S2 pocket was found to be the second most rigid pocket in thrombin upon ranking by global backbone flexibility. Pockets S3-S6 showed elevated global backbone flexibilities. The correlation between active site dynamics and specificity was quantified as Spearman rank correlation coefficient ρ. We found that global backbone dynamics correlate strongly to cleavage entropies (specificity) with ρ = 0.886.

Additionally, we quantified sub-pocket flexibility as backbone dihedral entropies, Sφ and Sψ from the distribution of dihedral angles φ and ψ during 1μs molecular dynamics simulation. It should be noted that cleavage entropy and backbone dihedral entropy are different concepts which should not be confused even though both rely on entropy. Whereas cleavage entropy measures specificity relying on substrate data via information entropy, backbone dihedral entropies capture local flexibility based on molecular dynamics data reflecting thermodynamic entropy.

Backbone dihedral entropy yields Spearman rank correlation coefficients of ρφ = 0.657 and ρψ = 0.886 to cleavage entropy. Mapping of dihedral entropies capturing local backbone flexibility to the binding site of thrombin are shown in Fig 2. Both employed metrics for local flexibility clearly demonstrate the interplay of local flexibility and specificity. Identification of P1-S1 interactions as main carrier of substrate specificity, followed by P2-S2 interactions, is in good agreement with experimental findings from literature [40].

**Fig 3:**
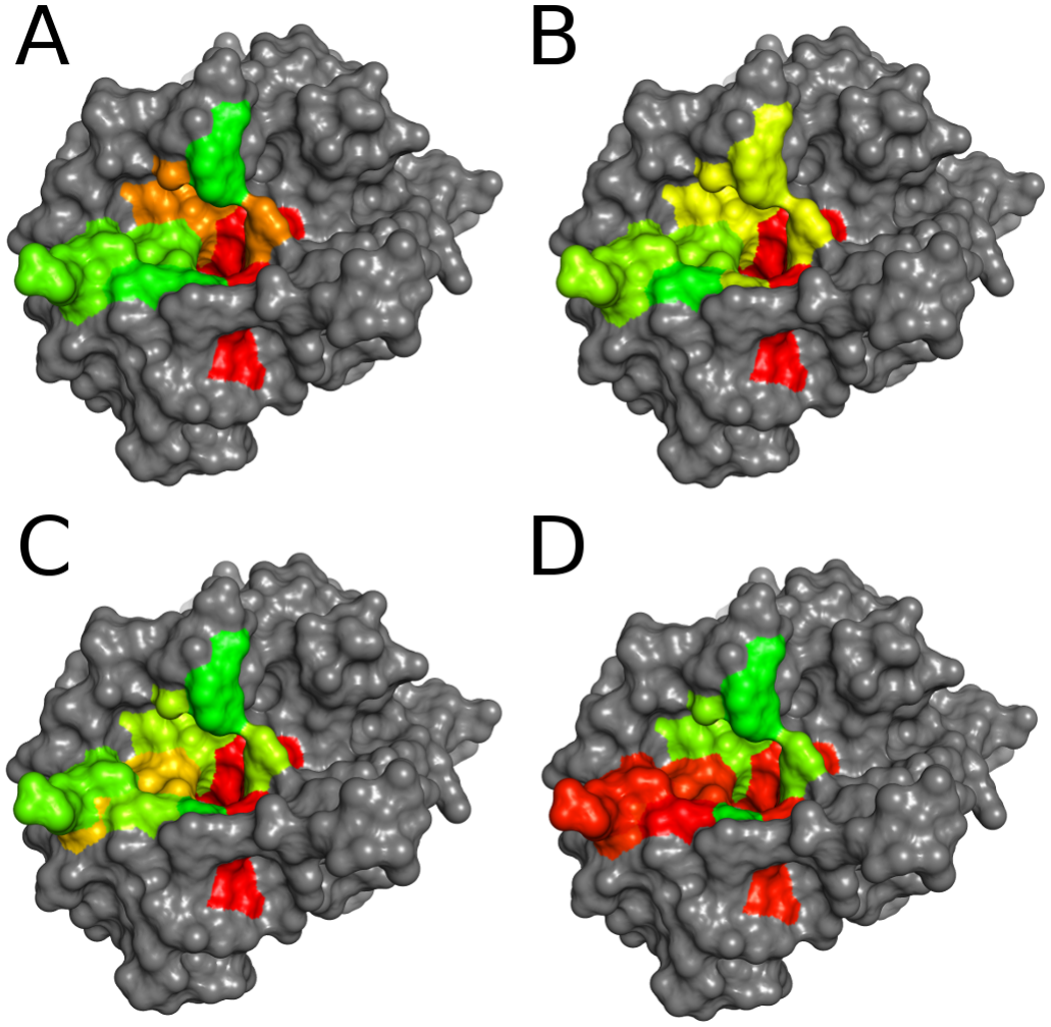
Water thermodynamics within the thrombin binding site in comparison with cleavage entropy. Cleavage entropy mapped to thrombin sub-pockets (A) with colors ranging from red (specific) to green (unspecific) in comparison with sub-pocket average translational entropy of water (B), orientational entropy of water (C) and solute-water enthalpic interactions (D) with colors ranging from red (high ordering and strong interactions) to green (low ordering and weak interactions). (B) and (C) show strong correlation with binding specificity (A), whereas enthalpic interactions with water are not as predictive (D). See Figure B in S1 File for a mapping based on ranks rather than absolute values.

In addition to protein dynamics, water is known to contribute to protein recognition [41]. Thus, we quantified water thermodynamics in thrombin binding pockets using Grid Inhomogeneous Solvation Theory (GIST) [42]. We found strong correlations of water ordering and binding specificity similar to the correlations described above between specificity and protein flexibility (see Fig 3). Seemingly protein rigidity extends in the form of water ordering into the first solvation shell and thus further contributes to specificity.

The rigid S1 pocket of thrombin contains highly ordered water molecules with entropies considerably lower than the bulk reference (see Table 2). Translational entropy increases for outer and more solvent-exposed sub-pockets. Orientational entropy contributions are all negative, thus water molecules are overall more ordered than in bulk. Correlations of water entropies to binding specificity are strong with ρ = 0.886 for orientational entropy and ρ = 0.600 for translational entropy.

**Table 2:** Sub-pocket-wise water thermodynamics for holo simulations: dTS_trans_, dTS_orient_, H_solv_ per water. The rigidity of sub-pocket S1 is reflected in a higher ordering of water molecules. Enthalpic interactions between solvent and solute are very similar within pockets S1, S4, S5, and S6.

	S6	S5	S4	S3	S2	S1
dTS_trans_ [kcal/mol]	0.11	0.36	0.12	-0.001	-0.052	-0.51
dTS_orient_ [kcal/mol]	-0.45	-0.53	-0.91	-0.22	-0.61	-1.38
H_solv_ [kcal/mol]	-10.0	-10.4	-9.69	-3.56	-5.30	-10.0

Explicit water molecules in molecular dynamics simulations are an excellent probe for enthalpic contributions to binding, as they do not only capture hydrogen bonding but also electrostatics and even non-polar interactions. In our analysis water enthalpy does not correlate to specificity to an extent comparable to water ordering measured by solvation entropy. Absolute enthalpic interactions between water and the protein identify the S1 pocket as most important anchor point but fail to rank other pockets in terms of specificity. A mean water-water interaction of -9.533 kcal/mol per water is expected for the TIP3P water model [43]. Despite, S2 and S3 pockets in fact repel waters binding from bulk. However, the S2 pocket is a major contribution to thrombin specificity. In consequence, Spearman correlation is only ρ = 0.200 between water enthalpies and cleavage entropy.

Several studies had hinted at qualitative correlations between protease dynamics and substrate specificity [44–48]. Our quantitative correlation of structural and dynamic data allows us to directly relate local flexibility with sub-pocket specificity of the protease thrombin. Correlations are derived from a ligand-free simulation and thereby support the theory that binding preferences are influenced by the intrinsic conformational ensemble of the receptor. Thus, specificity is following a paradigm of conformational selection.

**Fig 4:**
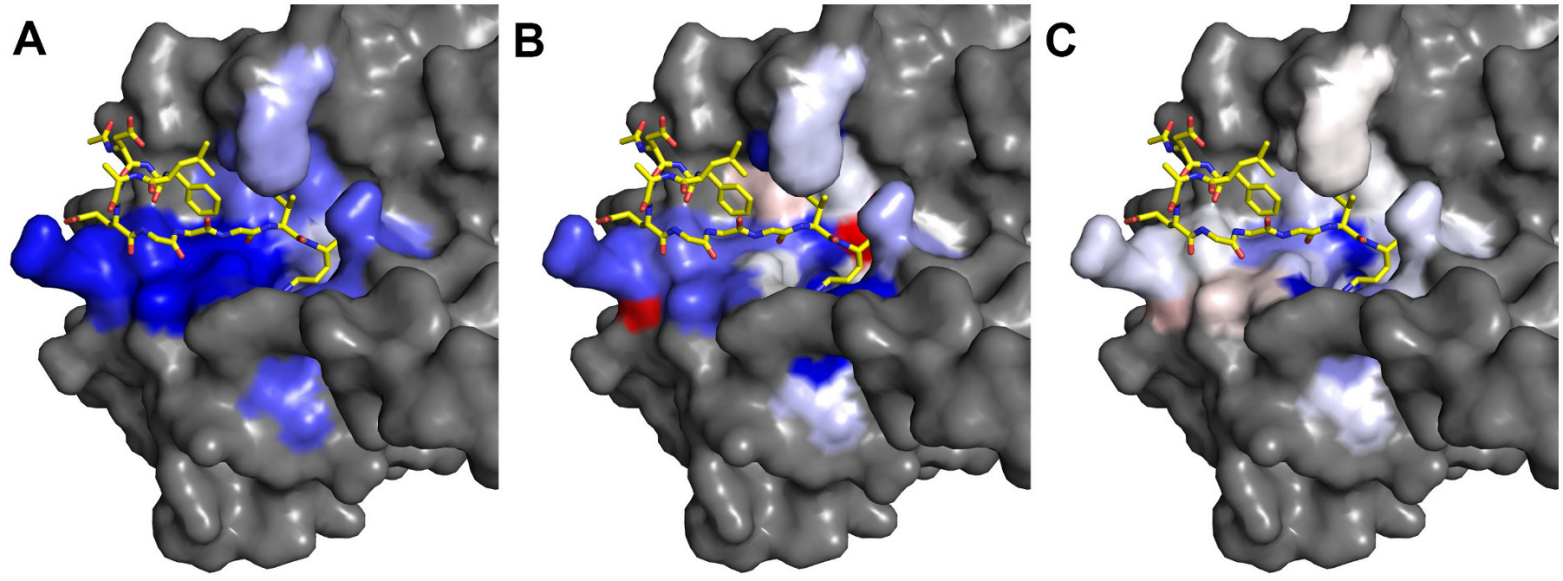
Dynamics changes upon fibrinogen binding to thrombin. Residue-wise mapping of flexibility difference maps Thr_com,Fib_—Thr_holo,Fib_ with colors ranging from blue (complex more rigid than holo) over white (no change) to red (complex more flexible than holo). Rigidification on substrate binding is shown for global backbone B-factors (A). For φ entropy (B) rigidification of some parts on complex formation is shown. A slight mobilization of Leu-99 is observed. For ψ entropy (C), no significant binding site rigidification apart from the S1 and S2 sub-pocket is shown.

Conformational selection is also confirmed when comparing simulations of holo and substrate-bound thrombin. Sampled conformational space for the substrate-bound form is a subset of the holo conformational ensemble. This is in agreement with studies on enzyme kinetics in literature [49,50,20]. The comparison of holo and complexed simulations revealed differences in active site dynamics upon substrate binding (see Fig 4). Global flexibility is reduced for all binding site residues in the simulated complex. Still, some residues oppose the overall trend to rigidification in Sφ: Leu-99 is mobilized by substrate binding. This is due to the hydrophobic contact of Leu-99 with Phe at P9 of the fibrinogen-derived peptide that mobilizes the backbone. As substrate peptides are bound to the catalytic cleft of proteases via distinct hydrogen bonding patterns, we investigated the hydrogen bonding characteristics of the thrombin-fibrinogen complex. In our simulations P1-S1 interactions are characterized by strong hydrogen bonding, forming on average more than three hydrogen bonds. Also S6-P6 interactions are mainly driven by hydrogen bonds, S3-P3 partially relies on hydrogen bonding of the backbone. Pockets S2, S4 and S5 show very little or no hydrogen bonding with the substrate peptide at all.

The most specific binding site region of thrombin, the S1 pocket, shows tightest hydrogen bonding (see Table 3). Nevertheless, the Spearman rank correlation coefficient ρ between hydrogen bond occupancy and cleavage entropy (specificity) is very low. Accordingly, ρ = -0.058 indicates an inverse correlation of hydrogen bond occupancy and cleavage entropy, where low values indicate specific substrate recognition. Still, the absolute value of ρ is close to zero and thus lower than correlation coefficients found for global backbone flexibility and dihedral entropy shown beforehand.

**Table 3:** Hydrogen bonding between thrombin and the bound peptide. In addition to P1-S1 interactions, also P6-S6 interactions rely on hydrogen bonding. The relatively specific S2 pocket does not show any hydrogen bonding at all.

	S6	S5	S4	S3	S2	S1
Occupancy	1.04	<0.01	0	0.59	0	3.37

Hence, local dynamics of the holo system provide more information about substrate specificity than hydrogen bonding in the complex. This shows that capturing enthalpic information only is not sufficient to explain the sub-pocket specificity of thrombin. Regions like the rigid S2 pocket in thrombin can be specific interaction partners even in absence of direct chemical interactions.

Additionally, we show a propagation of receptor dynamics into local water entropy. Rigid pockets show highly ordered water molecules. These highly ordered water molecules might be interpreted as probes of molecular interactions. Ordered water molecules imply well-defined exit vectors or particular pocket shapes and thus high specificity. Thereby, these ordered water molecules by specific binding reflect conformational selection from the hydration point of view. Similar correlations have already been described for PDZ domains [51], where the interplay of binding promiscuity and conformational selection is well accepted [52].

We observe very similar trends when analyzing a second thrombin complex with a factor XIII-derived peptide as well as a third complex formed by thrombin and a fragment of the protease-activated receptor 1 (see Table A and Table B in S1 File). Again, entropic contributions from binding site dynamics derived from microsecond scale simulations are more predictive for local sub-pocket specificity than enthalpic measures. The consistency of these trends observed for all three simulated systems strengthens our confidence that the correlations are caused by actual dynamic processes rather than sampling artifacts.

We demonstrate a tight interplay of flexibility and specificity in the substrate recognition of thrombin relying on conformational selection. Rigid binding site regions provide less diverse conformations, thus matching fewer binding partners. This in turn leads to specific substrate recognition. The described effect is independent of enthalpic considerations which we found insufficient to explain sub-pocket specificity of thrombin. In summary we find that active site flexibility plays a central role in substrate readout, turning local dynamics into a key player in the specificity of the protease thrombin. The paradigm “dynamics govern specificity” should be transferable to any biomolecular recognition process. Thus, it should provide guidance for finding accurate specific anchor points in structure-based drug design.

## Materials and Methods

### Data mining and cleavage entropy calculation

We used the protease substrate collection from MEROPS [13] to quantify protease specificity via calculation of cleavage entropy calculation [16]. Based on 185 substrates (database identifier: S01.217, accession date: 14.6.2013) we calculated sub-pocket-wise cleavage entropies for all substrate positions P6-P1 that are in direct contact with the thrombin binding site. Sub-pocket-wise cleavage entropies depict a metric for substrate specificity ranging from 0 for stringent substrate readout to 1 for random substrate binding.

### Preparation of the X-ray structures

Structures of protease-substrate complexes allow mapping of substrate specificity to the binding site of proteases. We based our study on the X-ray structure of thrombin in complex with fibrinogen (PDB: 1FPH [53]) occupying the non-prime side of the binding cleft. The structure contains a chloromethylketone-inhibited form of thrombin, where the substrate peptide is covalently linked to the active site residues Ser-195 and His-57. We removed the covalent bonds and added an N-methyl group (NME) to the C-terminal of the peptides to neutralize the free charge. Analogously, we capped the N-terminal of the substrate peptides with an acetyl group (ACE). This terminal group was already present in case of the complex with fibrinogen-derived decapeptide. Thus, the simulated complex represents a non-covalent protein-ligand complex.

We removed the light chains of thrombin as well as the hirudin chain. Water molecules assigned to the heavy chain were preserved, as buried water molecules are known be crucial for protease simulations [54,55]. Structures were protonated using the protonate3D protocol from MOE [56] and manually checked. This resulted in a simulation topology not containing any protonated histidines, while automatically assigned histidine tautomers were preserved.

To study conformational selection effects, we generated a holo system in addition to the complex structure. Therefore, we removed the bound fibrinogen from the binding site. This resulted in two topologies for molecular dynamics simulations: the original thrombin complex Thr_com,Fib_ as well as the respective holo system Thr_holo,Fib_. The constructed holo system is highly similar to a published apo structure of thrombin (PDB: 1HXF [57]) with a Cα RMSD as low as 0.4Å. We did not reprotonate the system after substrate removal to ensure comparability between both systems. Finally, we added solvent boxes of explicit TIP3P water [58] with a minimum wall distance of 12Å around the proteins using tleap from AmberTools [59].

Four additional systems were prepared for comparison: We created another simulation setup based on the crystal structure of thrombin with a bound factor XIII-derived peptide (PDB: 1DE7 [60]) resulting in a system for the complex as well as an apo system after deleting the peptide ligand. A third independent starting structure was built based on the co-crystal structure of thrombin and a bound protease-activated receptor 1 fragment (PDB: 3LU9 [61]). Again, we performed simulations for both complex and apo state modeled via deletion of the peptide ligand. Those additional systems represent the fast form of thrombin with bound Na^+^ ions. These ions were held in place over simulation time by applying flat bottom harmonic restraints to the coordinating protein atoms.

### Binding site definition

For the definition of the thrombin binding site, the complex structures of thrombin with bound fibrinogen and thrombin with bound factor XIII were used. All residues of thrombin with at least one atom within a radius of 3.5Å around the peptide substrates were considered as binding pockets S6-S1. Based on this definition a direct comparison between substrate data (P6-P1) and structure and dynamics data for the pockets S6-S1 is possible.

To obtain a more balanced binding site definition between both systems Thr_com,Fib_ and Thr_com,F13_, we unified both sets of residues leading to a common definition for the sub-pockets (see Table C in S1 File for a list of residues following the numbering scheme for chymotrypsinogen [62]).

### Molecular dynamics simulations

All atom simulations were performed using the GPU implementation of pmemd [63] in AMBER12 [59] using the AMBER forcefield 99SB-ILDN [64]. A uniform neutralizing plasma was applied to neutralize the box net charge [65]. We used a Van der Waals cutoff of 8Å, SHAKE algorithm [66] on hydrogen atoms allowed a time step of 2.0fs. Simulations were performed at 300K maintained by the Langevin thermostat [67].

After minimization with harmonic constraints on protein heavy atoms, the system was gradually heated to 300K in NVT ensemble. As last equilibration step an unrestrained density equilibration over 1ns was performed. For more details on the extensive equilibration protocol including restraint settings and details on heating and cooling steps see Wallnoefer et al. [54].

After equilibration 500 million unrestrained sampling steps were run, equivalent to a total simulation time of 1μs for each system. We saved 50,000 equally spaced snapshots to trajectory for later analysis. Real simulation time per system was approximately four weeks on single GeForce GTX 680 GPUs.

### Analysis of molecular dynamics simulations

Analyses of trajectories were carried out using ptraj and cpptraj [68] from AmberTools [59]. To check stability and convergence of our simulations we generated two-dimensional RMSD plots of Cα-atom positions in the binding site regions (see Table A in S1 File for a list of included residues).

The molecular dynamics simulation of unbound thrombin sampled a conformational space near the native X-ray structure with plateau RMSD values below 2.5Å for Cα positions of the binding site. The simulation of thrombin in complex with fibrinogen remained even closer to the input structure with RMSD values below 1.5Å. Two-dimensional RMSD plots between the holo and the complex simulation show that overlapping portions of conformational space were sampled. Removal of the substrates from the active site of thrombin did not significantly alter sampled configurations within the simulation time scale of 1μs, as Cα RMSD values between snapshots from both simulations remained below 3Å (see Figure C in S1 File for two-dimensional RMSD-plots).

Global flexibility in the protein was captured as residue-wise B-factors for Cα-atoms after a single global alignment. Apart from global alignments, flexibility metrics can be defined based on internal coordinates. The backbone dihedral angles φ and ψ define the local backbone conformation of a protein [69]. The distribution of these angles during the course of a molecular dynamics trajectory allows to infer a state probability function and subsequently calculate a local backbone entropy. Nonparametric kernel density estimation was applied in order to obtain a state probability distribution function as demonstrated previously [70]. To avoid a decrease of the kernel at boundaries when estimating the density, data around -180°/180° is periodically duplicated.

Integration according to Sα = —R ∫ p(α) ln p(α) dα yields thermodynamic entropy arising from conformational flexibility in the degree of freedom α [36]. This metric gives an upper bond for total backbone entropy, as correlation between the backbone angles is not accounted for due to the fact that a one-dimensional probability density function is obtained. Total thermodynamic entropy is lower as correlation of individual degrees of freedom restricts the overall conformational space. We calculated independent entropies for both dihedral angles φ and ψ as Sφ and Sψ. Ordered states correspond to low entropies. Therefore, a single dihedral peak with a width of 1° yields an entropy of zero (rigid region), whereas disordered states yield positive values (flexible region).

All residue-wise flexibility metrics were grouped into sub-pockets and arithmetic averages were calculated. Pocket-wise flexibilities were mapped to the starting structures and visualized using Pymol [71].

To support emerging correlations between binding site rigidity and substrate specificity, we performed a statistical analysis of the molecular dynamics trajectories by splitting them in ten parts (100ns each). The calculated global backbone flexibility (see Figure D in S1 File) and dihedral entropy (see Figures E and F in S1 File) all presented S1 and S2 as most rigid parts of the thrombin binding site. Pockets S3-S6 are more flexible and show varying rankings of these four sub-pockets.

We used Grid Inhomogeneous Solvation Theory to calculate hydration thermodynamics from simulation trajectories [72,42,73]. We extracted 5,000 equal-spaced snapshots from our trajectory for analysis on a grid with 6Å edge length and default 0.5Å spacing centered on the center of mass of respective sub-pocket residues.

To capture interactions between the substrate peptide and the protease, we extracted ensemble-averaged hydrogen bond occupancies from the complex simulations and grouped them into the respective protease sub-pockets. We applied default definitions for hydrogen bonds. Thus, the maximum heavy atom distance was set to 3.0Å, the maximum angle between donor, hydrogen and acceptor to 45°.

## Supporting Information

Rank-based binding site mappings, two-dimensional RMSD plots of simulations, trajectory splitting analyses for different flexibility metrics, a definition of the binding pockets, as well as detailed results for the simulation of the fibrinogen-derived thrombin complex and the PAR1-derived complex are available as Supporting Information.

## Supporting Information

S1 File Figure A: Rank-based flexibility landscapes of the thrombin binding site. Cleavage entropy mapped to thrombin sub-pockets (A) with colors ranging from red (specific) to green (unspecific) in comparison with sub-pocket average global backbone B-factors (B), φ entropy (C) and ψ entropy (D) with colors ranging from red (rigid or low dihedral entropy, respectively) to green (flexible or high dihedral entropy, respectively). Coloring is based on ranking of respective pockets. Figure B: Rank-based mapping of water thermodynamics to the thrombin binding site. Cleavage entropy mapped to thrombin sub-pockets (A) with colors ranging from red (specific) to green (unspecific) in comparison with sub-pocket average translational entropy of water (B), orientational entropy of water (C) and solute-water enthalpic interactions (D) with colors ranging from red (high ordering and strong interactions) to green (low ordering and weak interactions). Coloring is based on ranking of respective pockets. Figure C: 2D-RMSD plots of thrombin holo and fibrinogen bound (Cα positions of the binding site). 2D-RMSD plots of thrombin 1000ns molecular dynamics trajectory (A-C). Comparison of holo simulation (A) and complex simulation (B) shows higher RMSD values in the holo simulation indicating rigidification on substrate binding. Combination of (A) and (B) into (C) shows sampling of overlapping conformational space. Figure D: Trajectory splitting for global Backbone B Factors. Sub-pockets S1 and S2 consistently show lower B Factors than sub-pockets S3-S6. Higher B Factors in trajectory parts 6–9 indicate conformational changes within the binding site. Figure E: Trajectory splitting for Sφ. Sub-pockets S1 and S2 show the lowest φ entropies. Sub-pockets S3-S6 show varying higher φ entropies. Higher φ entropies in trajectory parts 6–9 indicate conformational changes within the binding site. Figure F: Trajectory splitting for Sψ. Again, sub-pockets S1 and S2 show the lowest ψ entropies. Sub-pockets S3-S6 show varying higher ψ entropies. Higher ψ entropies in trajectory parts 6–9 indicate conformational changes within the binding site. Table A: Analysis of the thrombin-F13 system. Sub-pocket-wise analysis of entropic and enthalpic metrics for the thrombin-F13 system. Here, 1μs trajectories of both complex and holo structure were prepared and analyzed in analogy to the system setup described for the thrombin-Fib system. We observe similar trends as for the main system. Entropic metrics (B-factors, dihedral entropies, hydration entropies) correlate to cleavage entropy, whereas enthalpic metrics (solute-water interactions, hydrogen bonding) show less correlation to substrate specificity. All binding site properties except the hydrogen bonding occupancies were derived from the apo simulation. Table B: Analysis of the thrombin-PAR1 system. Sub-pocket-wise analysis of entropic and enthalpic metrics for the thrombin-PAR1 system. Here, 1μs trajectories of both complex and holo structure were prepared and analyzed in analogy to the system setup described for the thrombin-Fib system. We observe similar trends as for the main system. Entropic metrics (B-factors, dihedral entropies, hydration entropies) correlate to cleavage entropy, whereas enthalpic metrics (solute-water interactions, hydrogen bonding) show less correlation to substrate specificity. All binding site properties except the hydrogen bonding occupancies were derived from the apo simulation. Table C: Thrombin sub-pocket residues. Sub-pocket residues were chosen according to proximity to the corresponding ligand binding site. All residues with at least one atom in a proximity of less than 3.5Å were included in the pocket definition. Pocket definitions for Thr_Com,Fib_ and Thr_Com,F13_ were merged to obtain a more generic binding site definition.
(PDF)
