# Supplementary material for: Dynamics Govern Specificity of a Protein-Protein Interface: Substrate Recognition by Thrombin
Source: PLoS One. 2015 Oct 23;10(10):e0140713. doi: 10.1371/journal.pone.0140713 (PMC4619833; doi:10.1371/journal.pone.0140713)
Supplement: S1 File — Figure A: Rank-based flexibility landscapes of the thrombin binding site. Cleavage entropy mapped to thrombin sub-pockets (A) with colors ranging from red (specific) to green (unspecific) in comparison with sub-pocket average global backbone B-factors (B), φ entropy (C) and ψ entropy (D) with colors ranging from red (rigid or low dihedral entropy, respectively) to green (flexible or high dihedral entropy, respectively). Coloring is based on ranking of respective pockets. Figure B: Rank-based mapping of water thermodynamics to the thrombin binding site. Cleavage entropy mapped to thrombin sub-pockets (A) with colors ranging from red (specific) to green (unspecific) in comparison with sub-pocket average translational entropy of water (B), orientational entropy of water (C) and solute-water enthalpic interactions (D) with colors ranging from red (high ordering and strong interactions) to green (low ordering and weak interactions). Coloring is based on ranking of respective pockets. Figure C: 2D-RMSD plots of thrombin holo and fibrinogen bound (Cα positions of the binding site). 2D-RMSD plots of thrombin 1000ns molecular dynamics trajectory (A-C). Comparison of holo simulation (A) and complex simulation (B) shows higher RMSD values in the holo simulation indicating rigidification on substrate binding. Combination of (A) and (B) into (C) shows sampling of overlapping conformational space. Figure D: Trajectory splitting for global Backbone B Factors. Sub-pockets S1 and S2 consistently show lower B Factors than sub-pockets S3-S6. Higher B Factors in trajectory parts 6–9 indicate conformational changes within the binding site. Figure E: Trajectory splitting for Sφ. Sub-pockets S1 and S2 show the lowest φ entropies. Sub-pockets S3-S6 show varying higher φ entropies. Higher φ entropies in trajectory parts 6–9 indicate conformational changes within the binding site. Figure F: Trajectory splitting for Sψ. Again, sub-pockets S1 and S2 show the lowest ψ entropies. Sub-pockets S3- [file pone.0140713.s001.pdf]

Supporting Information:

Dynamics Govern Specificity of a Protein-Protein Interface: Substrate Recognition by Thrombin

Julian E. Fuchs, Roland G. Huber, Birgit J. Waldner, Ursula Kahler, Susanne von Grafenstein, Christian Kramer, Klaus R. Liedl

Supplementary Materials:

Figures A-F

Table A-C

Figure A:

Rank-based flexibility landscapes of the thrombin binding site.

Cleavage entropy mapped to thrombin sub-pockets (A) with colors ranging from red (specific) to green (unspecific) in comparison with sub-pocket average global backbone B-factors (B), φ entropy (C) and ψ entropy (D) with colors ranging from red (rigid or low dihedral entropy, respectively) to green (flexible or high dihedral entropy, respectively). Coloring is based on ranking of respective pockets.

Figure B:

Rank-based mapping of water thermodynamics to the thrombin binding site.

Cleavage entropy mapped to thrombin sub-pockets (A) with colors ranging from red (specific) to green (unspecific) in comparison with sub-pocket average translational entropy of water (B), orientational entropy of water (C) and solute-water enthalpic interactions (D) with colors ranging from red (high ordering and strong interactions) to green (low ordering and weak interactions). Coloring is based on ranking of respective pockets.

Figure C:

2D-RMSD plots of thrombin holo and fibrinogen bound (Cα positions of the binding site).

2D-RMSD plots of thrombin 1000ns molecular dynamics trajectory (A-C). Comparison of holo simulation (A) and complex simulation (B) shows higher RMSD values in the holo simulation indicating rigidification on substrate binding. Combination of (A) and (B) into (C) shows sampling of overlapping conformational space.

Figure D:

Trajectory splitting for global Backbone B Factors.

Sub-pockets S1 and S2 consistently show lower B Factors than sub-pockets S3-S6. Higher B Factors in trajectory parts 6-9 indicate conformational changes within the binding site.

Figure E:

Trajectory splitting for Sφ.

Sub-pockets S1 and S2 show the lowest φ entropies. Sub-pockets S3-S6 show varying higher φ entropies. Higher φ entropies in trajectory parts 6-9 indicate conformational changes within the binding site.

Figure F:

Trajectory splitting for Sψ.

Again, sub-pockets S1 and S2 show the lowest ψ entropies. Sub-pockets S3-S6 show varying higher ψ entropies. Higher ψ entropies in trajectory parts 6-9 indicate conformational changes within the binding site.

Fig. A:

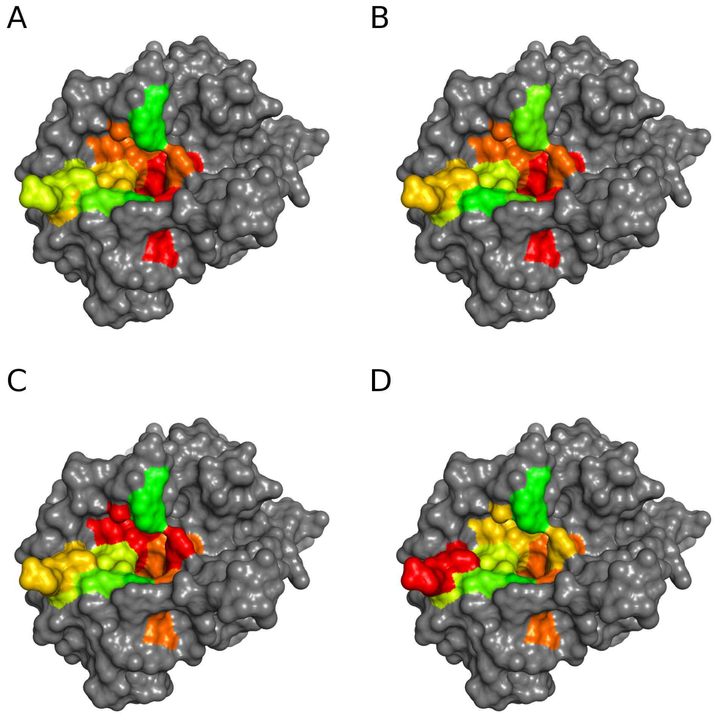

Fig. B:

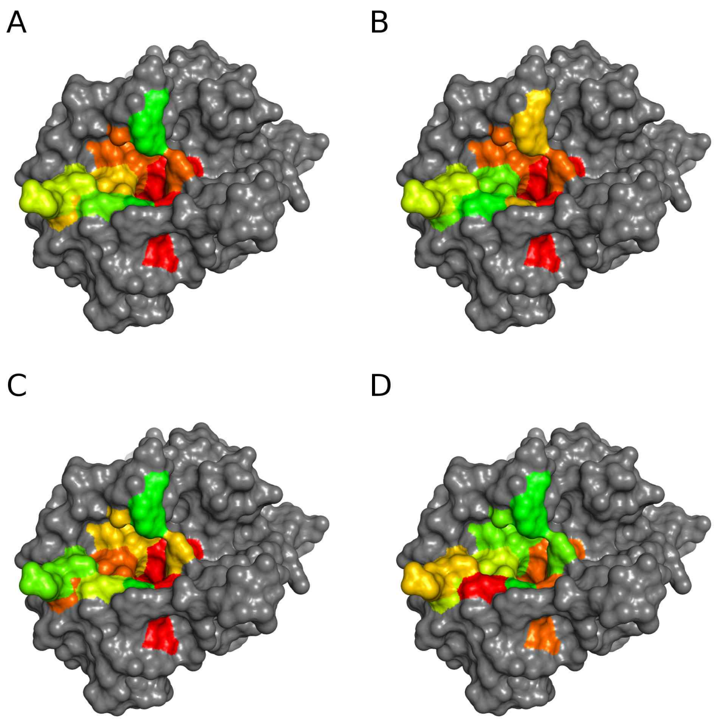

Fig. C:

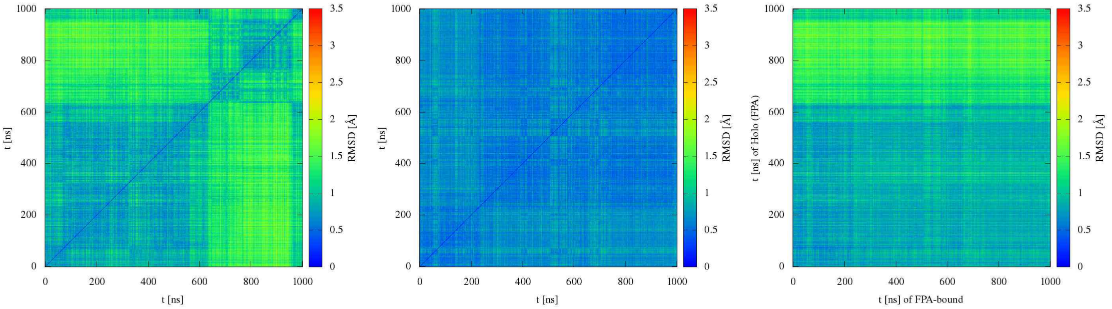

t [ns]
t [ns]
RMSD [Å]
t [ns]
t [ns]
RMSD [Å]
t [ns] of Holo (FPA)
t [ns] of FPA-bound
RMSD [Å]

Fig. D:

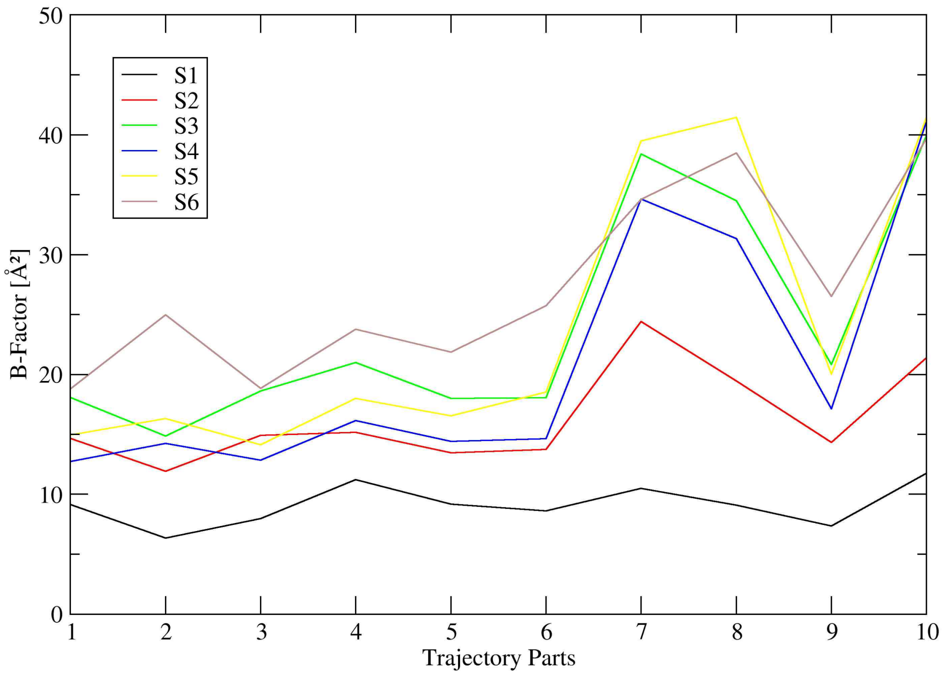
S1
S2
S3
S4
S5
S6
B-Factor [Å²]
Trajectory Parts
0
10
20
30
40
50
1
2
3
4
5
6
7
8
9
10

Fig. E:

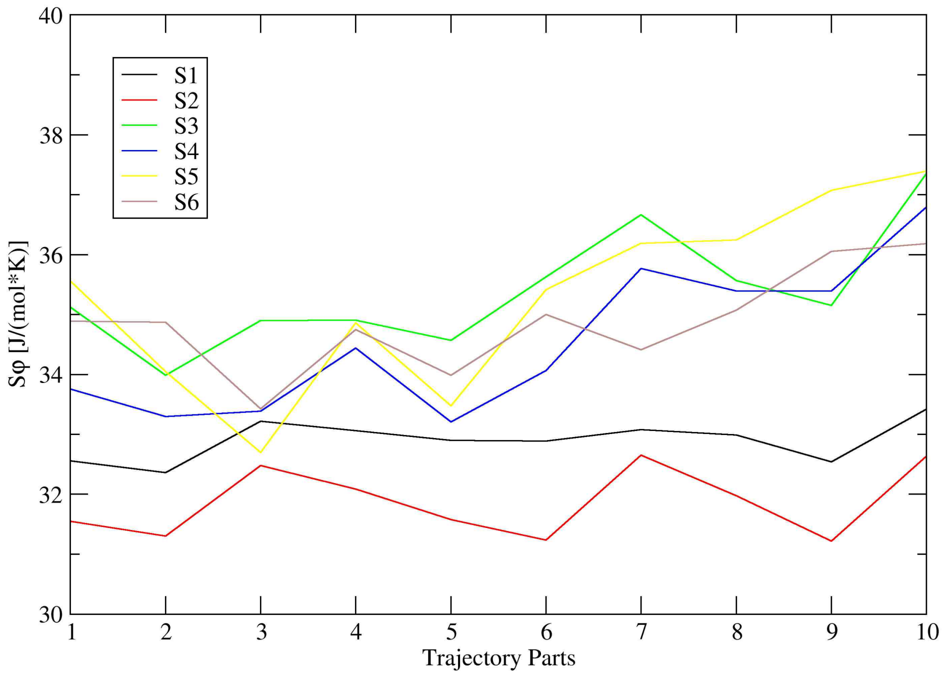

S1
S2
S3
S4
S5
S6
Sφ [J/(mol*K)]
Trajectory Parts
40
38
36
34
32
30
1
2
3
4
5
6
7
8
9
10

Fig. F:

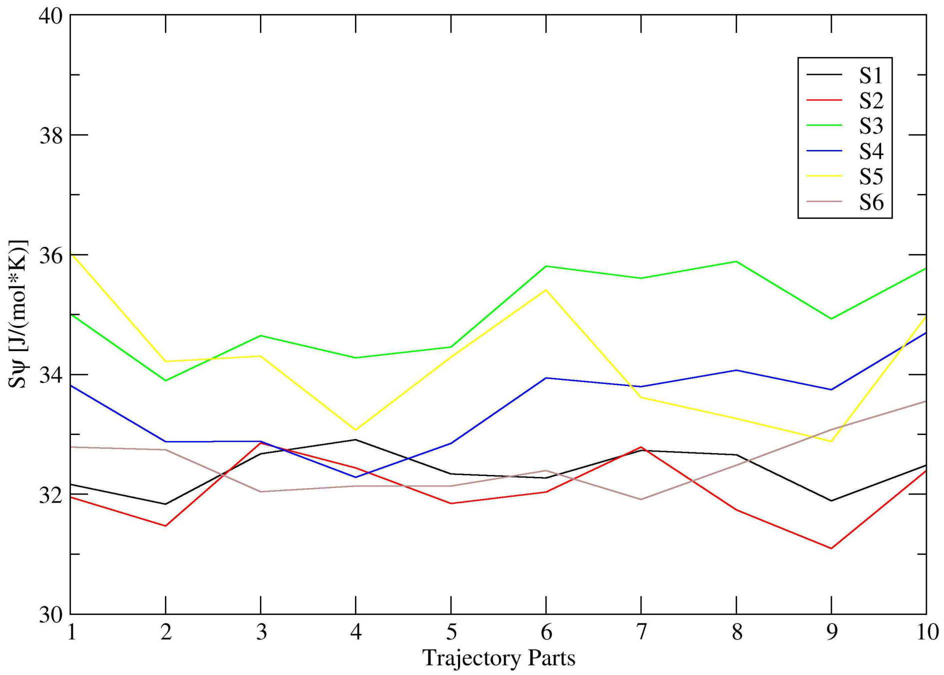

Sψ [J/(mol*K)]
40
38
36
34
32
30
1
2
3
4
5
6
7
8
9
10
Trajectory Parts
S1
S2
S3
S4
S5
S6

Table A: Analysis of the thrombin-F13 system.

Sub-pocket-wise analysis of entropic and enthalpic metrics for the thrombin-F13 system. Here, 1µs trajectories of both complex and holo structure were prepared and analyzed in analogy to the system setup described for the thrombin-Fib system. We observe similar trends as for the main system. Entropic metrics (B-factors, dihedral entropies, hydration entropies) correlate to cleavage entropy, whereas enthalpic metrics (solute-water interactions, hydrogen bonding) show less correlation to substrate specificity. All binding site properties except the hydrogen bonding occupancies were derived from the apo simulation.

	S6	S5	S4	S3	S2	S1	$r_{Spearman}$
$S_{Cleavage}$	0.899	0.959	0.892	0.971	0.635	0.176	
Global Cα B-Factor [Å²]	97.2	85.6	71.4	66.8	32.1	14.4	0.60
Sφ [J/(mol*K)]	36.3	38.4	37.3	37.6	33.7	33.5	0.89
Sψ [J/(mol*K)]	35.2	37.1	36.1	36.8	33.7	32.9	0.89
dTS_{trans} [kcal/mol]	0.12	0.15	0.11	0.07	0.10	-0.38	0.43
dTS_{orient} [kcal/mol]	-0.44	-0.38	-0.44	-0.18	-0.93	-1.10	1.00
H_{solv} [kcal/mol]	-8.24	-9.13	-8.30	-5.77	-7.36	-8.70	0.20
H-Bond Occupancy	0.39	0.07	0.27	0.02	0.00	4.02	0.37

Table B: Analysis of the thrombin-PAR1 system.

Sub-pocket-wise analysis of entropic and enthalpic metrics for the thrombin-PAR1 system. Here, 1µs trajectories of both complex and holo structure were prepared and analyzed in analogy to the system setup described for the thrombin-Fib system. We observe similar trends as for the main system. Entropic metrics (B-factors, dihedral entropies, hydration entropies) correlate to cleavage entropy, whereas enthalpic metrics (solute-water interactions, hydrogen bonding) show less correlation to substrate specificity. All binding site properties except the hydrogen bonding occupancies were derived from the apo simulation.

	S6	S5	S4	S3	S2	S1	$r_{Spearman}$
$S_{Cleavage}$	0.899	0.959	0.892	0.971	0.635	0.176	
Global Cα B-Factor [Å²]	47.6	30.7	27.9	32.6	20.4	13.2	0.83
Sφ [J/(mol*K)]	36.0	36.5	37.5	37.7	33.6	34.0	0.77
Sψ [J/(mol*K)]	34.1	37.8	36.1	36.6	32.7	32.8	0.77
dTS_{trans} [kcal/mol]	-0.12	-0.19	-0.74	-0.06	-0.19	-0.39	0.60
dTS_{orient} [kcal/mol]	-1.01	-1.14	-1.63	-0.22	-0.78	-0.94	0.09
H_{solv} [kcal/mol]	-11.69	-11.81	-11.37	-3.76	-6.29	-7.93	-0.09
H-Bond Occupancy	0.02	0.31	< 0.01	0.84	0.30	3.65	0.03

Table C: Thrombin sub-pocket residues.

Sub-pocket residues were chosen according to proximity to the corresponding ligand binding site. All residues with at least one atom in a proximity of less than 3.5Å were included in the pocket definition. Pocket definitions for $Thr_{Com,Fib}$ and $Thr_{Com,F13}$ were merged to obtain a more generic binding site definition.

Pocket	S6	S5	S4	S3	S2	S1
Residues	Arg-173	Ile-174	Thr-172	Trp-60D	His-57	His-57
	Ile-174	Glu-217	Ile-174	Trp-215	Tyr-60A	Asp-189
			Trp-215	Gly-216	Trp-60D	Ala-190
			Gly-216	Glu-217	Leu-99	Cys-191
			Glu-217	Gly-219	Glu-192	Glu-192
					Ser-214	Gly-193
					Trp-215	Asp-194
						Ser-195
						Val-213
						Ser-214
						Trp-215
						Gly-216
						Gly-219
						Cys-220
						Asp-221A
						Gly-226
						Phe-227
